# Protective Effects and Mechanisms of *N*-Phenethyl Caffeamide from UVA-Induced Skin Damage in Human Epidermal Keratinocytes through Nrf2/HO-1 Regulation

**DOI:** 10.3390/ijms20010164

**Published:** 2019-01-04

**Authors:** Yin Chu, Po-Yuan Wu, Chien-Wen Chen, Jia-Ling Lyu, Yi-Jung Liu, Kuo-Ching Wen, Chien-Yih Lin, Yueh-Hsiung Kuo, Hsiu-Mei Chiang

**Affiliations:** 1 Department of Cosmeceutics, China Medical University, Taichung 40402, Taiwan; dooo517@outlook.com (Y.C.); walnut0727@hotmail.com (C.-W.C.); rain26842006@gmail.com (J.-L.L.); ella8175@gmail.com (Y.-J.L.); kcwen0520@mail.cmu.edu.tw (K.-C.W.); 2 Department of Dermatology, China Medical University Hospital, Taichung 40402, Taiwan; wu.poyuan@gmail.com; 3 School of Medicine, China Medical University, Taichung 40402, Taiwan; 4 Ph.D Program for Biotechnology Industry, China Medical University, Taichung 40402, Taiwan; 5 Department of Biotechnology, Asia University, Taichung 41354, Taiwan; yihlin@asia.edu.tw; 6 Department of Chinese Pharmaceutical Sciences and Chinese Medicine Resources, China Medical University, Taichung 40402, Taiwan

**Keywords:** *N*-phenethyl caffeamide, photodamage, photoinflammation, heme oxygenase-1 (HO-1), nuclear factor erythroid 2–related factor 2 (Nrf2), 8-hydroxy-2’-deoxyguanosine (8-OHdG)

## Abstract

The skin provides an effective barrier against physical, chemical, and microbial invasion; however, overexposure to ultraviolet (UV) radiation causes excessive cellular oxidative stress, which leads to skin damage, DNA damage, mutations, and skin cancer. This study investigated the protective effects of *N*-phenethyl caffeamide (K36) from UVA damage on human epidermal keratinocytes. We found that K36 reduced UVA-induced intracellular reactive oxygen species (ROS) production and induced the expression of the intrinsic antioxidant enzyme heme oxygenase-1 (HO-1) by increasing the translocation of nuclear factor erythroid 2–related factor 2 (Nrf2). K36 could inhibit the phosphorylation of extracellular-signal-regulated kinase (ERK) and c-Jun N-terminal kinases (JNK) and reduce UVA-induced matrix metalloproteinase (MMP)-1 and MMP-2 overexpression; it could also elevate the expression of tissue inhibitors of metalloproteinases (TIMP). In addition, K36 ameliorated 8-hydroxy-2′-deoxyguanosine (8-OHdG) induced by UVA irradiation. Furthermore, K36 could downregulate the expression of inducible nitric oxide synthase (iNOS) and interleukin-6 (IL-6) and the subsequent production of nitric oxide (NO) and prostaglandin E_2_ (PGE_2_). Based on our findings, K36 possessed potent antioxidant, anti-inflammatory, antiphotodamage, and even antiphotocarcinogenesis activities. Thus, K36 has the potential to be used to multifunctional skin care products and drugs.

## 1. Introduction

Human skin is composed of three primary layers: the epidermis, dermis, and hypodermis. The epidermis is the outermost layer of the skin that acts as the first barrier against environmental hazards and helps prevent water loss. Furthermore, keratinocytes account for nearly 95% of all cells that form the epidermis [1,2].

Ultraviolet (UV) radiation is a major hazard to the skin. The UV spectrum consists of three wavelength ranges: UVA (320–400 nm), UVB (280–320 nm), and UVC (200–280 nm) [3]. Approximately 90–99% of the solar radiation that reaches Earth’s surface is UVA, which penetrates deeper into the skin than UVB [4,5]. Although UVA has lower energy than UVB, overexposure to UVA may still result in the generation of excess reactive oxygen species (ROS) that can attack DNA strands and lead to the formation of various oxidation products [6]. A critical biomarker of oxidative stress is 8-hydroxy-2′-deoxyguanosine (8-OHdG), which correlates with many types of cancer and degenerative disease [7]. Oxidation products may trigger intrinsic apoptosis of cells; in this process, cytochrome C is released, which activates downstream proapoptotic caspases, causing the apoptosis of cells [8,9].

When skin cells encounter a large amount of intracellular oxidative stress, a self-defense system is triggered. Nuclear factor erythroid 2-related factor 2 (Nrf2), a transcription factor, translocates into the nucleus, binds with the antioxidant response element (ARE), and then increases the production of phase II detoxifying enzymes, including heme oxygenase-1 (HO-1) and NAD(P)H:quinone oxidoreductase 1, which can protect cells from oxidative damage [10,11]. A high level of HO-1 protein can be induced by UVA irradiation in multiple cell lines and cause anti-inflammatory and antiapoptotic activities by capturing excessive free radicals [12].

Many receptors of cytokines and growth factors are activated in the skin after UV irradiation, resulting in the activation of mitogen-activated protein (MAP) kinases and then the regulation of the downstream expression of proteins, such as activator protein-1 (AP-1) and matrix metalloproteinases (MMPs) [13,14]. These cytokines are believed to be correlated with cellular aging and carcinogenesis. The main mechanism underlying the UV-induced inflammatory response in the skin is ROS overproduction that activates nuclear factor-kappa B (NF-κB), leading to the production of tumor necrosis factor (TNF)-α and interleukin (IL)-6; furthermore, this activates cyclooxygenase-2 (COX-2) and prostaglandins (PGs), which result in the skin becoming warm, red, and swollen [15]. After UVA irradiation, the mRNA of inducible nitric oxide (NO) synthase (iNOS) is upregulated, thereby inducing the overproduction of NO, which may lead to cellular apoptosis [16,17].

**Figure 1:**
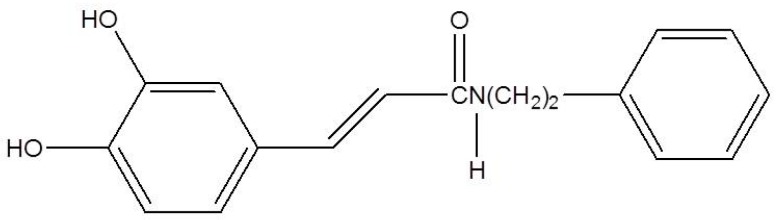
Structure of *N*-phenethyl caffeamide (K36).

*N*-phenethyl caffeamide (K36; Figure 1) is a derivative from caffeic acid phenethyl amide, which exhibits anti-diabetes, cardiovascular protective, and hepatoprotective potential [18,19]. In addition, K36 also shows potent antioxidant and free radical scavenging abilities [20]. Our previous study reported that K36 can reduce UVB damage by inhibiting type I procollagen degradation and stimulating collagen synthesis in human skin fibroblasts [21]. UVB is stronger than UVA while UVA is the major component of UV irradiation that reaches the earth’s surface. Therefore, this study was designed to investigate the reparative effect of K36 on UVA-induced oxidative damage and its signal transduction in human epidermal keratinocytes. In addition, the effect of K36 on UV-induced 8-OHdG and the regulation of protein overexpression related to UV damage in mouse dorsal skin were studied.

## 2. Results

### 2.1. Cytotoxicity of K36 on Keratinocytes

**Figure 2:**
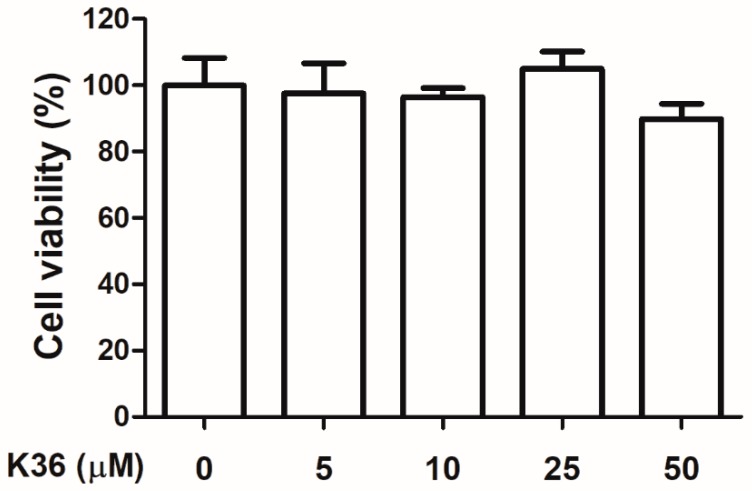
Cell viability (%) of human epidermal keratinocytes after various concentrations of K36 treatment. The bars and error bars present as mean ± SD (*n* = 8).

Human immortalized keratinocytes (HaCaT cells) were treated with various doses of K36, and the cell viability was measured using the 3-(4,5-dimethylthiazol-2-yl)-2,5-diphenyltetrazolium bromide (MTT) assay. The cell viabilities of Hs68 in K36 treatment groups exhibited no significant difference to the control group. The survival rate of 80% is a critical value of no cytotoxicity in many studies [22,23]. As shown in Figure 2, cell viability was over 80%, which indicated that K36 did not exhibit cytotoxicity.

### 2.2. Effect of UVA on Cell Viability and ROS Production

**Figure 3:**
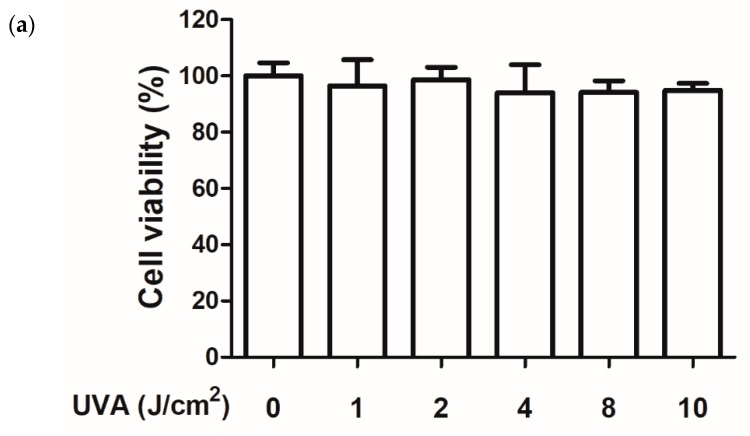

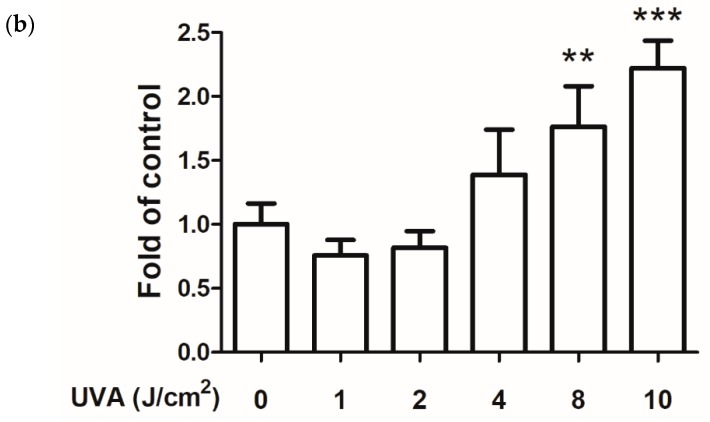
(**a**) Cell viability (%) in human epidermal keratinocytes after various UVA doses exposure. (**b**) Intracellular reactive oxygen species (ROS) level under various UVA doses in human epidermal keratinocytes; significant difference versus nonirradiated group: **, *p* < 0.01; ***, *p* < 0.001. The bars and error bars present as mean ± SD (*n* = 8).

HaCaT cells were exposed to various doses of UVA, and the intracellular ROS production of the cells was detected using 2′,7′-dichlorofluorescin diacetate (DCFDA) assays. After various doses of UVA irradiation, the cell viabilities of keratinocytes were all above 90% by MTT assay (Figure 3a). Subsequently, we performed a DCFDA assay to determine the ROS production induced by the various doses of UVA. Figure 3b shows that 10 J/cm^2^ of UVA significantly induced ROS production in keratinocytes to a 2.32-fold level compared with that in the control group without affecting cell viability. Therefore, 10 J/cm^2^ of UVA was selected for all subsequent experiments.

### 2.3. Antioxidant Effect of K36

**Figure 4:**
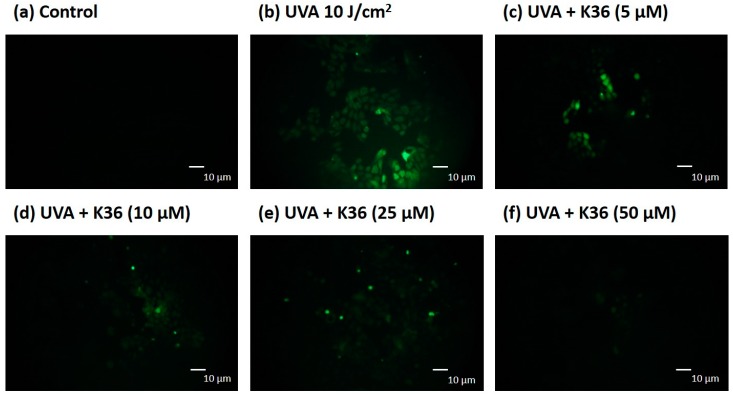

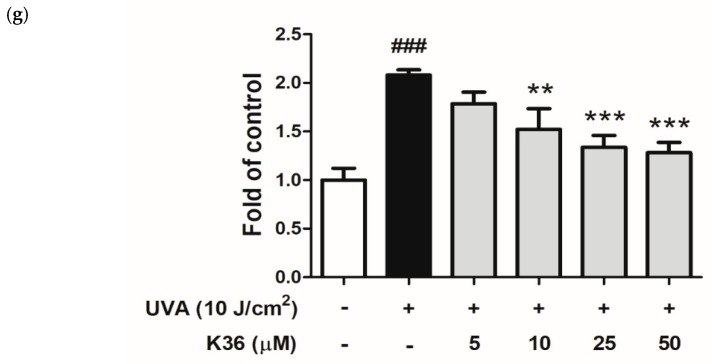
Effect of K36 on intracellular oxidative stress in UVA-irradiated human epidermal keratinocytes. The representative image of the effect of treatment on the cells (**a**–**f**) and the average value of the effects of triplicate experiment (**g**); significant difference versus nonirradiated group: ###, *p* < 0.001; significant difference versus irradiated and nontreatment group: **, *p* < 0.01; ***, *p* < 0.001. The bars and error bars present as mean ± SD.

We used the DCFDA assay to determine UVA-induced ROS production in keratinocytes. A representative image of the effect of UVA and K36 treatment on the cells is shown in Figure 4. The intracellular ROS production in UVA-irradiated keratinocytes was induced to a 2.08-fold level. After treatment with 5, 10, 25, and 50 μM of K36, the intracellular ROS content was significantly suppressed to 1.79, 1.52, 1.34, and 1.28-fold levels, respectively, compared with the control group. These results indicated that K36 inhibited UVA-induced ROS generation in a dose-dependent manner.

**Figure 5:**
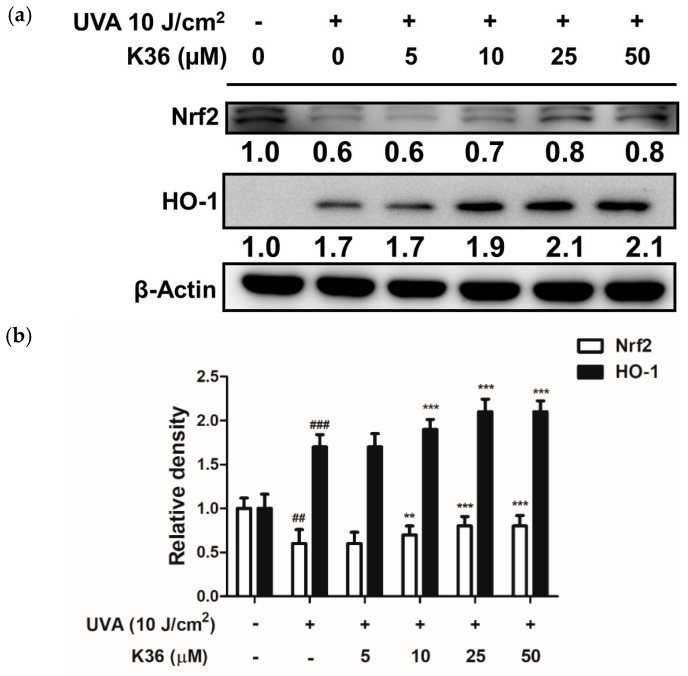
Effects of K36 on UVA-induced nuclear factor erythroid 2–related factor 2 (Nrf2) and heme oxygenase-1 (HO-1) expression in human epidermal keratinocytes. The representative image of the western blot (**a**) and the average value of the triplicate experiment (**b**); significant difference versus nonirradiated group: ##, *p* < 0.05; ###, *p* < 0.001; significant difference versus irradiated and nontreatment group: **, *p* < 0.01; ***, *p* < 0.001. The bars and error bars present as mean ± SD (*n* = 3).

We evaluated whether the antioxidant activity of K36 was related to the cellular self-defense system as well as to Nrf2 and its downstream enzymes. As shown in Figure 5, the Nrf2 protein expression of the UVA-irradiated keratinocytes was decreased to 0.6-fold of the control, and K36 treatment significantly restored the expression to 0.8-fold at a concentration of 50 μM. With regard to downstream protein expression, after UVA irradiation of 10 J/cm^2^, the expression of HO-1 increased to 1.7-fold compared with the control group and was also elevated after K36 treatment (Figure 5). These results indicated that K36 might protect keratinocytes from oxidative stress by inducing Nrf2 translocation and then increasing the expression of the antioxidant enzyme HO-1.

**Figure 6:**
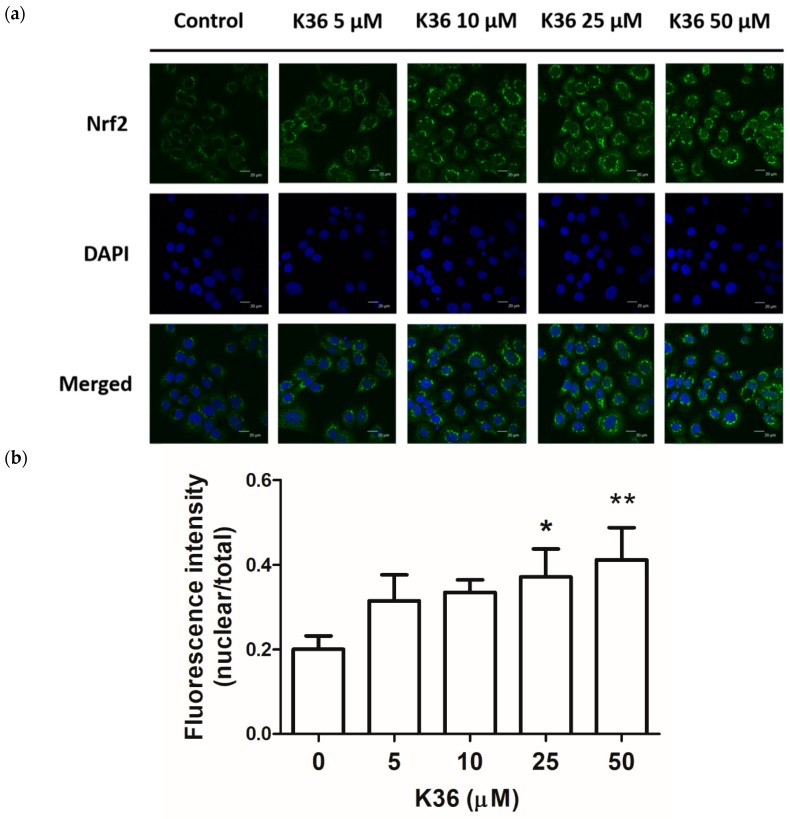
Effect of K36 on the translocation of Nrf2 in human epidermal keratinocytes. (**a**) The representative image of the effect of treatment on the cells and (**b**) The average value of nuclear/cytosol ratio of Nrf2 (*n* = 3). Significant difference versus control group: *, *p* < 0.05; **, *p* < 0.01. The bars and error bars present as mean ± SD.

To study the effect of K36 on the translocation of Nrf2, we employed immunofluorescence staining to observe whether K36 affects cellular Nrf2 translocation in keratinocytes. As shown in Figure 6, the nucleuses of the control group detected no Nrf2 via the immunofluorescence staining, while serial concentrations of K36 facilitated the translocation of Nrf2 into the nucleus and this effect was dose-dependently upregulated. 

### 2.4. Antiphotodamage Effect of K36

After being irradiated by UV radiation, various cytokines and receptors of growth factors are activated, leading to the activation and elevated expression of MAP kinases. Subsequently, AP-1, which is composed of c-Fos and c-Jun, translocates into the nucleus and regulates downstream protein expression, such as that of MMPs, which causes degradation of the extracellular matrix (ECM) [24,25]. Therefore, K36 was applied to investigate the effect on MAP kinase, AP-1, and MMP expression.

**Figure 7:**
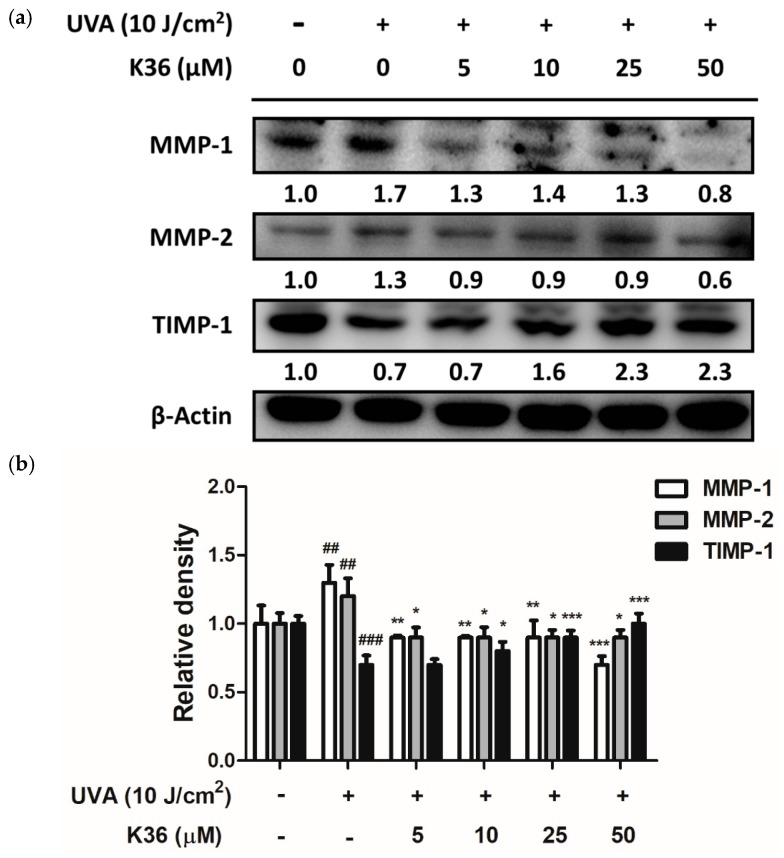
Effect of K36 on UVA-induced matrix metalloproteinase (MMP)-1, MMP-2, and tissue inhibitor of metalloproteinase (TIMP)-1 expression in human epidermal keratinocytes. The representative image of the western blot (**a**) and the average value of the triplicate experiment (**b**) (*n* = 3); significant difference versus nonirradiated group: ##, *p* < 0.01; ###, *p* < 0.001; significant difference versus irradiated and nontreatment group: *, *p* < 0.05; **, *p* < 0.01; ***, *p* < 0.001. The bars and error bars present as mean ± SD.

**Figure 8:**
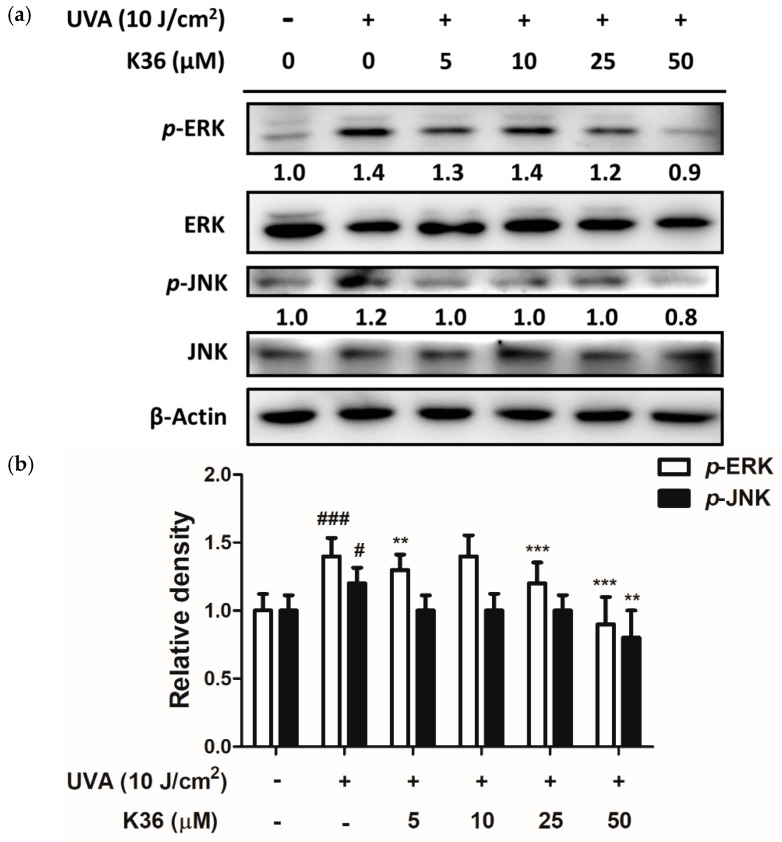
Effect of K36 on UVA-induced phosphorylation of ERK and JNK expression in human epidermal keratinocytes. The representative image of the western blot (**a**) and the average value of the triplicate experiment (**b**) (*n* = 4); significant difference versus nonirradiated group: #, *p* < 0.05; ###, *p* < 0.001; significant difference versus irradiated and nontreatment group: **, *p* < 0.01; ***, *p* < 0.001. The bars and error bars present as mean ± SD.

After 10 J/cm^2^ of UVA irradiation, the expression of both MMP-1 and MMP-2 protein was elevated to a 1.3-fold level compared with the control group and respectively downregulated to 0.7 and 1.1-fold levels after treatment with 50 μM of K36 (Figure 7). Furthermore, the endogenous MMP inhibitor TIMP-1 decreased to 0.7-fold compared with the control group after UVA irradiation; it was significantly restored back to a normal level after K36 treatment. Figure 8 shows that *p*-ERK and *p*-JNK expressions were induced to 1.4- and 1.2-fold levels, respectively, compared with the control group after 10 J/cm^2^ of UVA irradiation; furthermore, treatment with 50 μM of K36 could effectively reduce their expression to 0.9 and 0.8-fold levels, respectively.

**Figure 9:**
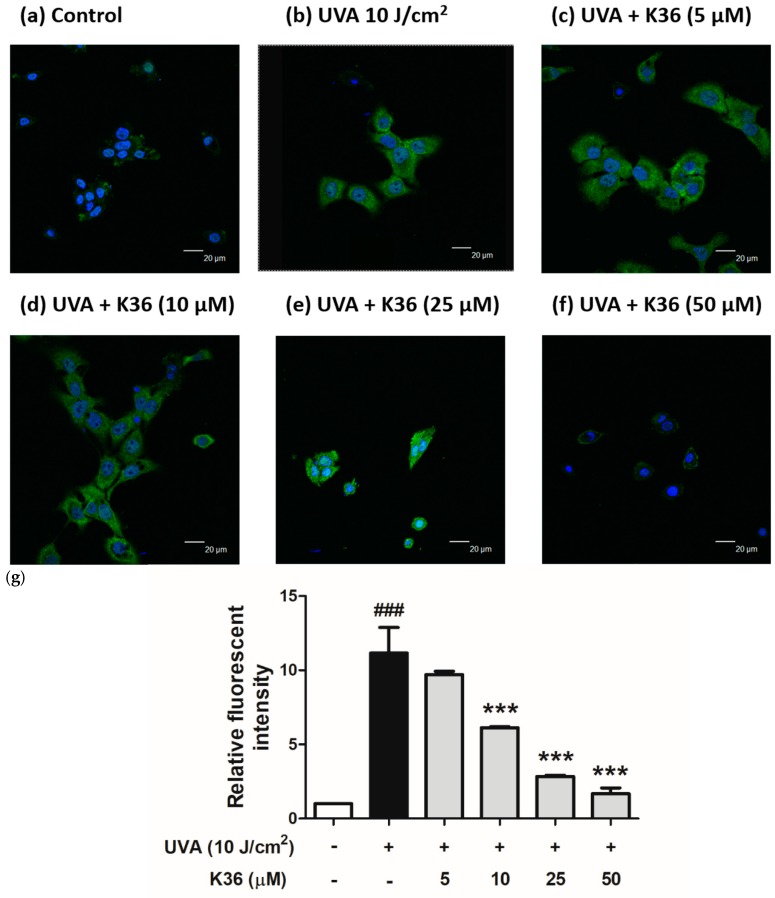
Effect of K36 on UVA-induced 8-hydroxy-2′-deoxyguanosine (8-OHdG) expression in human epidermal keratinocytes. The representative image of the effect of treatment on the cells (**g**) and the average value of the effects of triplicate experiment (**a**–**f**); significant difference versus nonirradiated group: ###, *p* < 0.001; significant difference versus irradiated and nontreatment group: ***, *p* < 0.001. The bars and error bars present as mean ± SD. White column is non UVA-irradiation and non K36 treated group, black column is UVA-irradiation and non K36 treated group, and grey column is UVA-irradiation with various concentration of K36 treatment group.

**Figure 10:**
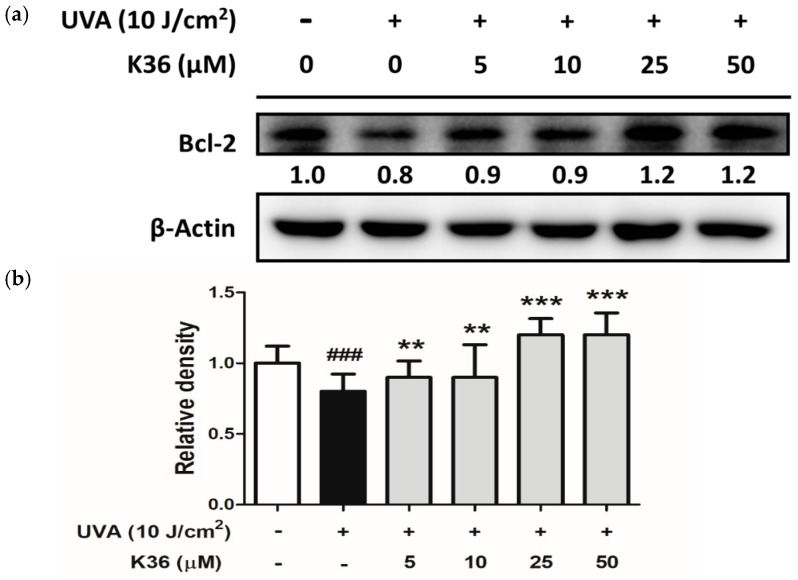
Effect of K36 on UVA-induced Bcl-2 expression in human epidermal keratinocytes. The representative image of the western blot (**a**) and the average value of the triplicate experiment (**b**); significant difference versus nonirradiated group: ###, *p* < 0.001; significant difference versus irradiated and nontreatment group: **, *p* < 0.01; ***, *p* < 0.001. The bars and error bars present as mean ± SD. White column is non UVA-irradiation and non K36 treated group, black column is UVA-irradiation and non K36 treated group, and grey column is UVA-irradiation with various concentration of K36 treatment group.

Excessive ROS induced by UVA irradiation causes tissue injury; DNA lesions then lead to apoptosis and skin cancer [26]. DNA present in nuclei and mitochondria is vulnerable to oxidative stress, which may produce the most mutagenic DNA lesion 8-OHdG [27,28]. We used immunofluorescence staining to observe whether K36 alleviates the production of UVA-induced 8-OHdG. After irradiation with 10 J/cm^2^ of UVA, 8-OHdG was induced, which was subsequently inhibited by K36 treatment (Figure 9). In addition, B-cell lymphoma 2 (Bcl-2) expression was decreased after exposure to 10 J/cm^2^ of UVA; however, K36 increased Bcl-2 expression in HaCaT cells (Figure 10). The results indicated that K36 may protect the skin cell from UVA-induced DNA damage and apoptosis.

### 2.5. Anti-Inflammatory Effect of K36

**Figure 11:**
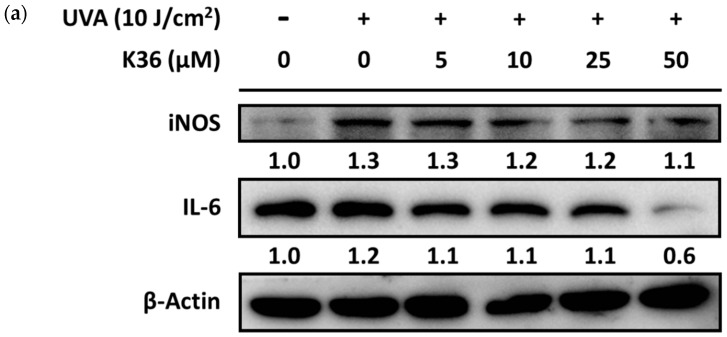

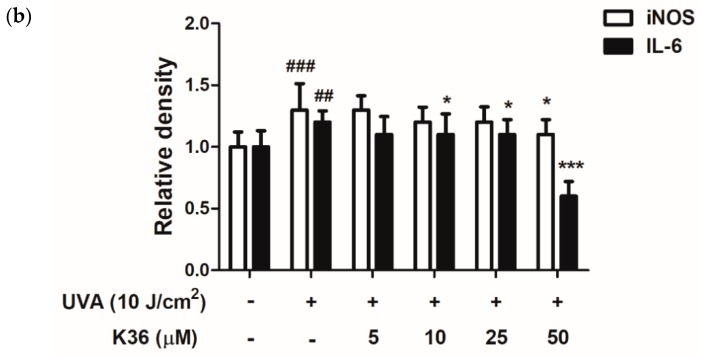
Effect of K36 on UVA-induced inducible nitric oxide (NO) synthase (iNOS) and IL-6 expression in human epidermal keratinocytes. The representative image of the western blot (**a**) and the average value of the triplicate experiment (**b**); significant difference versus nonirradiated group: ##, *p* < 0.01; ###, *p* < 0.001; significant difference versus irradiated and nontreatment group: *, *p* < 0.05; ***, *p* < 0.001. The bars and error bars present as mean ± SD.

After UVA irradiation, the expression of iNOS protein in keratinocytes was induced to a 1.3-fold level compared with the control group. After treatment with serial concentrations of K36, iNOS expression was reduced to at most a 1.1-fold level (Figure 11). IL-6 expression was elevated to a 1.2-fold level by 10 J/cm^2^ of UVA irradiation and suppressed to a 0.6-fold level by 50 μM of K36.

**Figure 12:**
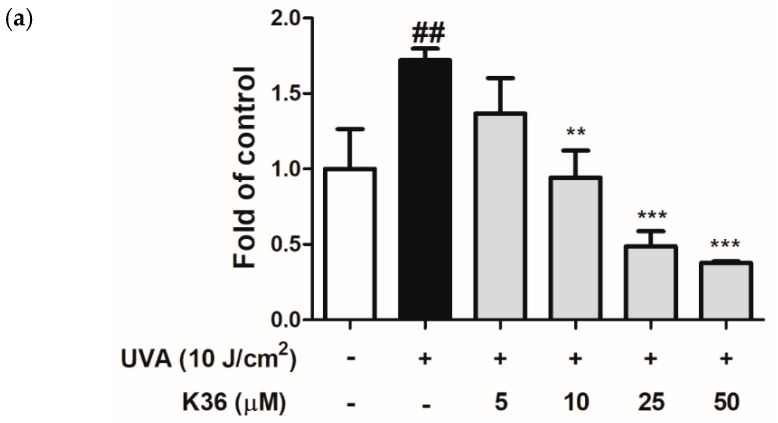

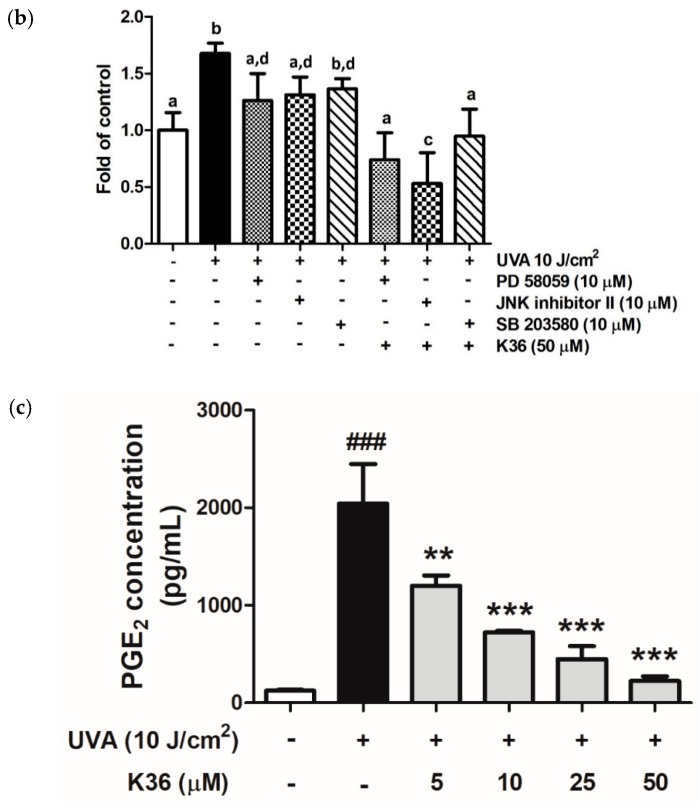
Effects of K36 on (**a**) NO production, (**b**) NO production after mitogen-activated protein (MAP) kinase inhibitors treatment and (**c**) PGE_2_ production in human epidermal keratinocytes (*n* = 8), respectively; significant difference versus nonirradiated group: ##, *p* < 0.01; ###, *p* < 0.001; significant difference versus irradiated and nontreatment group: **, *p* < 0.014; ***, *p* < 0.001. a–d: Values not followed by a common letter are significantly different. The bars and error bars present as mean ± SD (*n* = 8). White column is non UVA-irradiation and non K36 treated group, black column is UVA-irradiation and non K36 treated group, and grey column is UVA-irradiation with various concentration of K36 treatment group.

Nitric oxide is a paracrine regulator of various biological functions, such as vasodilation, neurotransmission, and cellular proliferation modification [29]; however, excessive NO production induced by cytokines is also implicated in tissue injury in inflammatory diseases [30]. Figure 12a shows that UVA exposure induced cellular NO production to a 1.7-fold level compared with the control group, and after treatment with 5–50 μM of K36, the NO level was remarkably reduced to 1.4, 0.9, 0.5, and 0.4-fold levels compared with the control group. To confirm whether the inhibition of K36 on NO is caused by MAP kinase, MAP kinase inhibitors were used to examine the effects of K36 on NO production. Inhibition of ERK phosphorylation by pretreatment with PD98059 resulted in the inhibition of NO production. The JNK inhibitor II results resembled those of ERK inhibitors, but not p-38 phosphorylation inhibitors (SB 203580). Cotreatment with K36 and MAP kinase inhibitors significantly decreased NO production (Figure 12b). The results indicated that K36 inhibited UVA-induced NO production by suppressing MAP kinase.

Our studies have shown that the prostaglandin E_2_ (PGE_2_) level in the keratinocyte cell culture medium was induced from 120.9 ± 9.3 to 2093.1 ± 396.6 pg/mL after UVA irradiation (Figure 12b). Furthermore, K36 treatment could reduce PGE_2_ production to 223.4 ± 47.0 pg/mL at 50 μM in a dose-dependent manner.

## 3. Discussion

Overexposure to UVA causes lipid, DNA, and protein damage in the skin through the generation of excessive ROS [31]. Excessive ROS can trigger many diseases or disorders, such as aging, DNA mutation, and cancer. Therefore, many studies have proven that substances capable of confronting oxidative stress are potential candidates for antiaging and anticancer treatment [28,32].

Our previous study found that K36 could inhibit the degradation of the ECM in skin dermal fibroblasts and reduce UVB-induced wrinkles in BALB/c hairless mice. However, the epidermis is the first barrier against environmental hazards and is mostly composed of keratinocytes. UV overirradiation may cause solar keratosis in normal keratinocytes; these abnormal cells can cause excessive ROS generation and then evolve into squamous cell carcinoma (SCC) [33]. Therefore, discovering an active ingredient that can protect skin cells from harmful effects of UV is critical.

Among all cellular self-defense systems, HO-1, regulated by Nrf2 and ARE, is one of the most crucial antioxidant pathways. Caffeic acid phenethyl ester was found to be able to induce HO-1 in HepG2 cells and may possess antioxidant and anti-inflammatory properties [34]. The results of this study indicated that K36 could effectively upregulate HO-1 protein expression in keratinocytes and induce Nrf2 translocation. Therefore, K36 may help keratinocytes to fight against oxidative stress through promoting Nrf2 translocation into nuclei and then elevating HO-1 expression.

MMPs are a group of endogenous proteases that are related to cell proliferation, ECM modification, and cell migration [35]. MMP-1, 2, and 9 are highly expressed in the skin after UV irradiation. MMP-1 mainly breaks down type I and III collagen, whereas MMP-2 breaks down gelatin and type IV collagen [36]. Moreover, MMP-2 and MMP-9 are crucial for angiogenesis and are possibly correlated with tumor growth [37]. TIMPs regulate the activity of MMPs and the balance between the activities of TIMPs and MMPs are closely related to the physiological and pathological processes characterized by the accumulation and degradation of the ECM [38]. TIMPs inhibit the proteolytic activity of MMPs directly to minimize extracellular matrix degradation [39]. As shown in Figure 7, K36 could effectively suppress the expression of UVA-induced MMP-1 and MMP-2 and restore the expression of TIMP-1 protein. It could result in the accumulation of ECM to prevent the UV-induced degradation of collagen.

Long-term exposure to UV induces ROS generation and causes DNA damage in the skin. Moreover, 8-OHdG can be induced by singlet oxygen, peroxynitrite, and hydroxyl radical; therefore, it is considered a critical marker of DNA damage caused by oxidative stress [40]. We evaluated whether K36 treatment could alleviate the production of 8-OHdG in keratinocytes after UVA irradiation. Figure 9 shows that 8-OHdG expression was dramatically increased after UVA irradiation and was inhibited after K36 treatment. The results indicated that the antioxidative activity of K36 may help in protecting the skin from UVA-induced DNA damage. ROS can attack DNA strands and lead to the formation of various oxidation products, which may trigger intrinsic apoptosis of cells [6,9]. In this study, K36 increased UVA-induced Bcl-2 expression and prevented skin cells from apoptosis.

Inflammation is an immune reaction that occurs in reaction to bodily injury; signs include elevated cellular metabolism, increased blood flow, and vasodilatation [41]. Overproduction of inflammatory cytokines can lead to cell aging and even to apoptosis. Caffeic acid phenethyl ester was reported to be able to inhibit NF-κB, COX-2, and IL-6 expression, indicating that it might have an anti-inflammatory effect [42]. Our results showed that K36 treatment could effectively reduce UVA-induced iNOS and IL-6 protein expression as well as the overexpression of the downstream inflammatory cytokines NO and PGE_2_, which indicates that K36 may have strong anti-inflammatory properties.

## 4. Materials and Methods

### 4.1. Materials

K36 was synthesized by the author, Professor Yueh-Hsiung Kuo [21], and was dissolved in dimethyl sulfoxide (DMSO) for experimentation. The final concentration of DMSO was lower than 0.05% and did not cause cytotoxicity. The Bradford reagent was purchased from Bio-Rad Laboratories (Hercules, CA, USA). DCFDA, DMSO, leupeptin, and phenylmethylsulfonyl fluoride were purchased from Sigma Chemical Co. (St. Louis, MO, USA). Sodium dodecyl sulfate (SDS), IGEPAL CA-630, MTT, Tris, and Tween20 were obtained from USB Corporation (Cleveland, OH, USA). Fetal bovine serum (FBS), penicillin–streptomycin, trypsin–EDTA, and Dulbecco’s Modified Eagle’s Medium (DMEM) were purchased from Gibco-Invitrogen (Carlsbad, CA, USA). TIMP-1 (GTX112096), IL-6 (GTX22105), HO-1 (GTX101147), and rabbit IgG antibody were purchased from Genetex (Beverly, MA, USA). ERK-1 (sc-93), MMP-1 (sc-12348), MMP-2 (sc-13595), Bcl-2 (sc-7382), c-Fos (sc-7202), COX-2 (sc-19999), JNK (sc-46006), and β-Actin (sc-1616) were purchased from Santa Cruz Biotechnology (Santa Cruz, CA, USA). A western blot detection reagent and PageRuler prestained protein ladder were purchased from Amersham Biosciences (Little Chalfont, Buckinghamshire, England). All other chemicals used in this study were of reagent grade.

### 4.2. Cell Culture and UVA Irradiation

Immortal human epidermal keratinocytes (HaCaT) were received as a kind gift from Dr. Louiz Chao (Department of Cosmeceutics, China Medical University, Taichung, Taiwan). HaCaT cells were maintained in DMEM supplemented with 10% FBS, 100 U/mL of penicillin, and 100 U/mL of streptomycin in a humidified atmosphere of 5% CO_2_ at 37 °C. The cell culture medium was removed, and cells were washed twice with phosphate-buffered saline (PBS). The cells were respectively irradiated with UVA at 0, 1, 2, 4, 8, and 10 J/cm^2^. UVA irradiation was performed using UV Crosslinkers XLE-1000A with a major wavelength of 365 nm (Spectroline, Algonquin, IL, USA). The cells were exposed to UV light directly and the intensity of UV irradiation was detected by a solarmeter (UVP, Upland, CA, USA). The distance between the UV light source and the sample was 15.2 cm. Subsequently, the cells were incubated in serum-free DMEM with or without various concentrations of K36 for 24 h in a humidified atmosphere of 5% CO_2_ at 37 °C.

### 4.3. Cell Viability Test

The MTT assay was performed to evaluate cell viability. After the cells were treated for 24 h, the MTT solution was added and then converted to insoluble formazan crystals. SDS-HCl (10%) was used to dissolve formazan, and its absorbance was determined at 570 nm using a spectrophotometer (Tecan, Grödig, Austria) [21,43].

### 4.4. Intracellular ROS Level

To measure the scavenging ability of K36 on intracellular ROS production in HaCaT cells, the cells were seeded into 24-well plates and irradiated with UVA. After treatment with serial concentrations of K36 for 3 h, DCFDA solution was added. The fluorescence intensity was determined at an excitation wavelength of 488 nm and an emission wavelength of 520 nm by using a fluorescence microplate reader (Thermo Electron Corporation, Vantaa, Finland) [44,45].

### 4.5. Western Blot Analysis

Protein expression was measured using western blotting as previously described with some modifications [21,46]. Cells were harvested, washed after the indicated treatments, and then lysed. The total protein from these cells was extracted and then separated on SDS-polyacrylamide gels using electrophoresis and detected using specific antibodies. β-Actin was used as an internal loading control. The immobilized proteins were then transferred onto polyvinylidene difluoride membranes and probed using antibodies. Immunoreactive proteins were detected using the ECL western blotting detection system (Fujifilm, LAS-4000, Tokyo, Japan), and signal strengths were quantified using a densitometric program (MultiGauge V2.2).

### 4.6. Immunofluorescence Staining

Immunofluorescence staining was measured as previously described with some modifications [46]. The cells were collected, fixed with 4% paraformaldehyde, and blocked with 5% nonfat milk containing 0.3% Triton X-100/PBS. After incubating with the primary antibody, the cells were incubated with Alexa Fluor 488 antirabbit IgG secondary antibody (Invitrogen, USA). The unbound secondary antibody was removed using PBS. Thereafter, the samples were counterstained with the ProLong Gold antifade reagent with DAPI and observed using a confocal laser scanning microscope (Leica DMIL, Munchen, Germany). The visual scoring of the Nrf2 translocation and 8-OHdG expression on each slide was based on the characterization of 100 randomly selected cells. The intensity of fluorescence of Nrf2 was quantified by using Image J.

### 4.7. NO Production

Quantitation of NO production in the culture medium was determined following the protocol provided by the supplier of the Grises reagent (Promega, Madison, WI, USA). The cell culture medium was collected, and sulfanilamide and NED solution were sequentially added to it. The absorbance was measured within 15 min in a plate reader with a filter at 540 nm [47].

### 4.8. PGE_2_ Production

PGE_2_ levels in the culture medium were measured using the protocol provided by the manufacturer of the kit (Cayman, Ann Arbor, MI, USA) with some modifications [46]. The cell culture medium was collected and added to a 96-well plate coated with the goat polyclonal antimouse IgG second antibody. Subsequently, the samples were added with the PGE_2_ first antibody and AchE tracer. Absorbance was measured using a filter at 420 nm after the addition of Ellman’s reagent.

### 4.9. Data Analysis

All data in this study are expressed as the mean ± standard deviation of independent experiments performed in triplicate for in vitro study. The statistical analysis of differences in means for each experimental group was performed using one-way analysis of variance followed by a Tukey post hoc test to detect intergroup differences; *p* < 0.05 was considered statistically significant.

## 5. Conclusions

**Figure 13:**
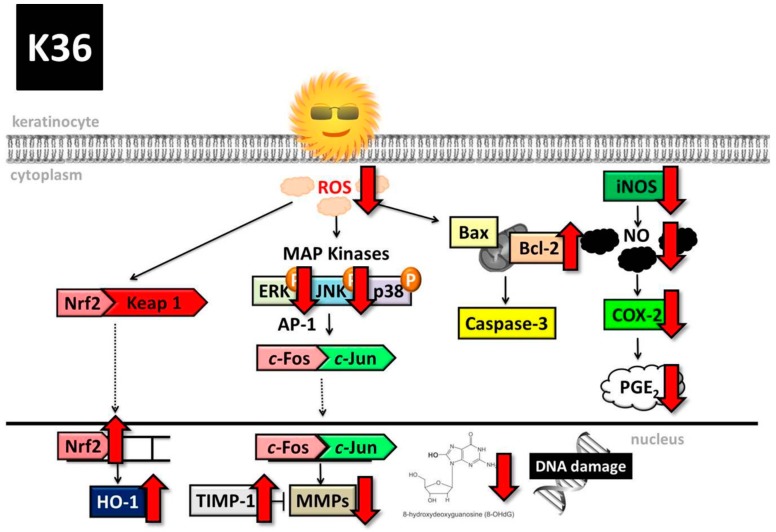
Schematic diagram showing effects of K36 on UVA-induced photodamage. COX-2 is cyclooxygenase-2, PGE_2_ is prostaglandin E_2_.

On the basis of all the aforementioned results, K36 has excellent antioxidant, antiphotodamage, and anti-inflammatory biological properties; therefore, it may possess high potential for being developed as a functional ingredient to protect skin from UV-induced damage (Figure 13).

## Abbreviations

AP-1	activator protein-1
CAPE	caffeic acid phenethyl ester
DCFDA	2′,7′-dichlorofluorescin diacetate
DMEM	Dulbecco’s Modified Eagle’s Medium
DMSO	dimethyl sulfoxide
ECM	extracellular matrix
K36	*N*-phenethyl caffeamide
MAP	kinase mitogen-activated protein kinase
MMPs	matrix metalloproteins
MTT	3-(4,5-dimethylthiazol-2-yl)-2,5-diphenyltetrazolium bromide
ROS	reactive oxygen species
SDS	sodium dodecyl sulfate
UV	ultraviolet
